# Comparative Evaluation of the In Vitro Antibacterial Activity of Preliminary Plant Preparations Against Phenotypically Multidrug‐Resistant Urinary Isolates of *Escherichia Coli*

**DOI:** 10.1155/bmri/1850504

**Published:** 2026-09-30

**Authors:** Mohammad Ahmadian, Mohammad Javad Mehdipour Moghaddam, Davood Bakhshi

**Affiliations:** ^1^ Department of Biology University Campus 2 University of Guilan Rasht Iran guilan.ac.ir; ^2^ Department of Biology Faculty of Sciences University of Guilan Rasht Iran guilan.ac.ir; ^3^ Department of Horticultural Science Faculty of Agricultural Sciences University of Guilan Rasht Iran guilan.ac.ir

**Keywords:** antimicrobial activity, essential oil, medicinal plants, multidrug-resistant *Escherichia coli*, urinary tract infection

## Abstract

**Background:**

Urinary tract infections (UTIs) caused by multidrug‐resistant (MDR) *Escherichia coli* pose an increasing therapeutic challenge due to rising antibiotic resistance and limited treatment options. This study evaluated the antibacterial activity and phytochemical composition of plant preparations from *Froriepia subpinnata* and *Eryngium caucasicum* against MDR urinary *E. coli* isolates.

**Methods:**

A total of 91 MDR *E. coli* isolates recovered from urine samples were examined. The antibacterial activities of the essential oil of *Froriepia subpinnata* (EoFs), the ethanolic extract of *Froriepia subpinnata* (EtFs), the ethanolic extract of *Eryngium caucasicum* (EtEc), the aqueous extract of *Froriepia subpinnata* (AqFs), and the aqueous extract of *Eryngium caucasicum* (AqEc) were assessed using agar well diffusion and broth microdilution assays to determine inhibition zones, minimum inhibitory concentrations (MIC), and minimum bactericidal concentrations (MBC). RP‐HPLC and GC–MS analyses were performed to conduct preliminary phytochemical profiling of the plant preparations and to generate a tentative chromatographic fingerprint of the essential oil, respectively.

**Results:**

The isolates exhibited extensive resistance to the tested antibiotics. Among the plant‐derived preparations, EoFs showed the broadest descriptive antibacterial profile. Across the tested concentration range, EoFs inhibited 49 of 91 isolates (54%). At the highest tested concentration, inhibition and killing were observed in 25 (27%) and 15 (16%) isolates, respectively. The overall descriptive activity pattern was EoFs > EtFs > EtEc > AqFs > AqEc. MIC and MBC distributions differed significantly among the five preparations (Friedman′s test, *p* = 3.28 × 10^−7^ and *p* = 1.37 × 10^−7^, respectively), although Kendall′s *W* values indicated small overall effect magnitudes (*W* = 0.098 and *W* = 0.103, respectively). Holm‐adjusted pairwise comparisons showed lower MIC and MBC values for EoFs than for several other preparations. RP‐HPLC showed retention‐time correspondence with catechin and quercetin in some preparations, whereas no comparable peak for chlorogenic acid was observed under the conditions used. GC–MS provided a qualitative chromatographic fingerprint of EoFs, with NIST spectral‐library comparisons used for descriptive characterization.

**Conclusion:**

*F. subpinnata* essential oil showed the strongest and most consistent antibacterial activity against MDR urinary *E. coli*. Although these findings highlight the potential of EoFs for further research, they do not establish clinical efficacy, a defined mechanism of action, or therapeutic equivalence to conventional antibiotics.

## 1. Introduction

Urinary tract infections (UTIs) are among the most common bacterial infections worldwide and pose a major public health concern because of their high incidence, recurrence, and rising antimicrobial resistance. Uropathogenic *Escherichia coli* (UPEC) accounts for most community‐acquired and nosocomial UTIs and remains the predominant etiological agent of uncomplicated urinary infections [1, 2]. In recent years, the rapid emergence of multidrug‐resistant (MDR) *E. coli* strains has posed a major challenge to effective treatment, reducing the efficacy of commonly used antibiotics and leading to increased healthcare costs and therapeutic failure [2, 3]. This alarming trend has been documented in recent global surveillance reports, which show a critical rise in resistance to first‐line agents [4, 5]. Consequently, the search for novel antimicrobial agents from natural sources has attracted increasing scientific attention.

Medicinal plants have long been recognized as important sources of bioactive compounds with diverse pharmacological properties. Plant‐derived secondary metabolites, including phenolic compounds, flavonoids, alkaloids, terpenoids, and essential oils, have been widely reported to exhibit antimicrobial, antioxidant, and anti‐inflammatory activities [6–8]. Several studies have demonstrated that plant extracts and essential oils can inhibit the growth of pathogenic bacteria, including antibiotic‐resistant gram‐negative organisms, suggesting their potential as alternative or complementary antimicrobial agents [8–10]. Recent literature from 2021–2025 further highlights the synergistic potential of these natural products and their volatile constituents as promising alternatives to combat MDR clinical isolates [11–14]. The complex chemical composition of essential oils may contribute to their antibacterial activity; however, the contribution of individual constituents and the risk of resistance development require experimental evaluation.

The Apiaceae family comprises more than 3000 species distributed worldwide and includes many aromatic and medicinal plants widely used in traditional medicine and the food industry [15]. Among these plants, species of the genus *Eryngium* have attracted considerable attention for their traditional medicinal uses and diverse phytochemical constituents [16]. Several studies have reported the presence of biologically active compounds, including terpenoids, saponins, flavonoids, and essential oils, in *Eryngium* species, which may contribute to their antimicrobial and antioxidant activities [16–19]. *Eryngium caucasicum*, a native plant widely distributed in northern Iran, is commonly consumed as a vegetable and flavoring agent and has also been used in traditional medicine to treat various ailments [17, 20]. Recent studies have continued to investigate the therapeutic benefits and bioactive profiling of this genus, confirming its value in modern phytotherapy [21, 22].

*Froriepia subpinnata*, another member of the Apiaceae family, grows primarily in the northern provinces of Iran, including Guilan, Mazandaran, and Golestan [23, 24]. This plant has traditionally been used for its diuretic, sedative, and antispasmodic effects [25]. *F. subpinnata* has been documented to possess antioxidant and antibacterial properties, which may be attributed to its phenolic compounds and volatile constituents [26]. Phytochemical investigations have identified various monoterpenes and sesquiterpenes in the essential oil of *F. subpinnata*, some of which have been linked to antimicrobial activity in other plant systems [27–29]. Despite this potential, limited research has evaluated its antibacterial efficacy against MDR urinary isolates. A comprehensive evaluation of various preparations—including essential oil, ethanolic extract, and aqueous extract—against a large clinical panel of MDR urinary isolates has not yet been conducted. The present study addresses this gap by investigating 91 clinical MDR urinary isolates and assessing the antibacterial activity of five plant preparations.

Therefore, the present study is aimed at evaluating the antibacterial activity of the essential oil of *F. subpinnata*, as well as the ethanolic and aqueous extracts of *F. subpinnata* and *E. caucasicum*, against 91 clinical MDR *E. coli* isolates from UTIs.

## 2. Materials and Methods

### 2.1. Bacterial Isolates

A total of 138 morning midstream urine specimens were collected from patients with suspected UTIs referred to the Razi Pathobiology Laboratory in Rasht, Iran. The samples were cultured using standard microbiological procedures, and 112 specimens yielded bacterial growth. Urine specimens were cultured on MacConkey agar and blood agar (Merck, Germany) and incubated aerobically at 37°C for 18–24 h. Among the positive cultures, 91 phenotypically MDR urinary *E. coli* isolates were selected for final analysis. Presumptive identification was based on colony morphology and Gram staining, and final confirmation was performed using biochemical tests, including catalase, oxidase, indole production, methyl red, citrate utilization, urease, and triple sugar iron agar tests [29]. Each isolate was obtained from a separate patient and was considered a distinct strain based on its antimicrobial susceptibility profile. The isolates were obtained from routine diagnostic laboratory samples and were anonymized before analysis; no patient‐identifiable information was available to the researchers. All confirmed isolates were preserved in tryptic soy broth (TSB) containing 20% glycerol at −80°C until further analysis.

### 2.2. Ethics Approval and Consent to Participate

The study protocol was approved by the Ethics Committee of the University of Guilan (Approval ID: IR.GUILAN.REC.1405.089). Human urine samples were obtained from routine diagnostic laboratory specimens and anonymized prior to analysis. The researchers had no access to patient‐identifiable information, and the ethics committee granted a waiver of informed consent given the study′s retrospective and anonymous nature. The study was conducted in accordance with the ethical principles of the Declaration of Helsinki.

### 2.3. Antibiotic Susceptibility Testing

Antimicrobial susceptibility testing was performed on 91 *E. coli* isolates collected and tested between May and July 2015. The disk diffusion method was performed according to the guidelines of the Clinical and Laboratory Standards Institute (CLSI) [30]. Bacterial suspensions were adjusted to a 0.5 McFarland turbidity standard (≈1.5 × 10^8^ CFU/mL) and uniformly inoculated onto Mueller–Hinton agar (MHA) plates. A panel of antibiotic disks was tested, including: amoxicillin (AMX, 25 *μ*g), ampicillin (AMP, 10 *μ*g), oxacillin (OXA, 5 *μ*g), piperacillin (PIP, 100 *μ*g), amoxicillin–clavulanic acid (AMC, 20/10 *μ*g), fosfomycin (FOF, 200 *μ*g), tetracycline (TE, 30 *μ*g), chloramphenicol (CHL, 30 *μ*g), streptomycin (STR, 10 *μ*g), ciprofloxacin (CIP, 5 *μ*g), cephalothin (CEF, 30 *μ*g), cefixime (CFM, 5 *μ*g), ceftazidime (CAZ, 30 *μ*g), ceftriaxone (CRO, 30 *μ*g), cefotaxime (CTX, 30 *μ*g), cefepime (FEP, 30 *μ*g), imipenem (IMP, 10 *μ*g), and meropenem (MEM, 10 *μ*g). Zone diameters were measured after incubation at 37^∘^C for 18–24 h and interpreted as susceptible (S), intermediate (I), or resistant based on the CLSI M100‐S25 (2015) interpretive criteria for *E. coli*. For antibiotics lacking official CLSI interpretive criteria for *E. coli* (e.g., OXA), results are presented descriptively. Quality control was performed using *E. coli* ATCC 25922. Isolates resistant to at least one agent in three or more of the following antimicrobial classes were classified as MDR: beta‐lactams (penicillins, cephalosporins, carbapenems), beta‐lactam/beta‐lactamase inhibitor combinations, aminoglycosides, fluoroquinolones, macrolides, TEs, and phenicols [31]. For FOF disk testing, the disks were confirmed to contain 200 *μ*g and were supplemented with glucose‐6‐phosphate per CLSI guidelines for urinary *E. coli* testing. The primary purpose of this testing was to characterize resistance patterns and confirm the MDR status of the isolates.

### 2.4. Preparation of Aqueous and Ethanolic Extracts

The aerial parts of *F. subpinnata* and *E. caucasicum* were collected during the flowering season from Lahijan (coordinates: 37°12 ^′^ N, 50°00 ^′^ E) and Rasht (coordinates: 37°16 ^′^ N, 49°35 ^′^ E) counties in Guilan province, Northern Iran. The plant materials were authenticated by Dr. Davood Bakhshi, a horticulture specialist at the University of Guilan. Voucher specimens were deposited in the Herbarium of the University of Guilan under Voucher Numbers APF54075 for *F. subpinnata* and APE53281 for *E. caucasicum*. The collected materials were thoroughly washed with distilled water, shade‐dried at room temperature (25°C ± 2°C) for 7–10 days until a constant moisture content of approximately 8.3% (for *F. subpinnata*) and 8.7% (for *E. caucasicum*) was reached, and then ground to a fine powder using a laboratory grinder.

Aqueous extracts were prepared by hot extraction. Briefly, 50 g of dried plant powder was mixed with 500 mL of distilled water and heated at 80°C for 30 min with continuous stirring. After cooling, the mixture was filtered through Whatman No. one filter paper, and the filtrate was concentrated under reduced pressure using a rotary evaporator at 40°C. The extraction yields of the aqueous extracts of *F. subpinnata* and *E. caucasicum* were calculated to be 11.4% and 12.6% (*w*/*w*), respectively, based on the initial dry weight.

Ethanolic extracts were prepared by maceration with slight modifications [32]. Briefly, 100 g of powdered plant material was soaked in 70% ethanol at a 1:10 (*w*/*v*) ratio and incubated at room temperature for 72 h with intermittent shaking. The mixture was filtered through Whatman No. one filter paper, and the solvent was removed using a rotary evaporator at 40°C under reduced pressure. The resulting crude ethanolic extracts of *F. subpinnata* and *E. caucasicum*, with yields of 7.8% and 8.2% (*w*/*w*), respectively, were dried and stored at 4°C in sterile, light‐protected glass vials for no longer than 4 weeks before use.

### 2.5. Essential Oil Extraction

Essential oils were extracted from the dried aerial parts of the plants by hydrodistillation with a Clevenger‐type apparatus, following the European Pharmacopeia method [33]. Briefly, 100 g of dried plant material was hydrodistilled for 3 h. The extraction yield for the essential oil of *F. subpinnata* was 0.6% (*v*/*w*) based on the dry weight of the plant material. The essential oils were then dried over anhydrous sodium sulfate and stored in sealed, dark glass vials at 4°C until further analysis.

The essential oil of *F. subpinnata*, ethanolic extracts of *F. subpinnata* and *E. caucasicum*, and aqueous extracts of *F. subpinnata* and *E. caucasicum* are referred to as EoFs, EtFs, EtEc, AqFs, and AqEc, respectively.

### 2.6. Agar Well Diffusion Assay

The antibacterial activity of aqueous and ethanolic extracts, as well as essential oils, was evaluated using the agar well diffusion method, as previously described [34]. Bacterial suspensions of the 91 phenotypically MDR urinary *E. coli* isolates were adjusted to a 0.5 McFarland standard and evenly swabbed onto MHA plates. Wells measuring 6 mm in diameter were prepared using a sterile cork borer. A 50 *μ*L volume of each tested dilution was added to the wells. The dilutions ranged from 10^−1^ to 10^−7^, prepared from a nominal 11.11 mg/mL stock solution of each plant preparation in 5% (*v*/*v*) DMSO. The preparations were allowed to prediffuse for 10 min before incubation. CHL disks (30 *μ*g) were tested in parallel as the positive control, whereas 5% (*v*/*v*) DMSO served as the negative solvent control. The plates were incubated at 37°C for 24 h, after which the inhibition‐zone diameters were measured in millimeters. No inhibition zone was observed around the negative solvent‐control wells.

### 2.7. Determination of Minimum Inhibitory Concentration (MIC) and Minimum Bactericidal Concentration (MBC)

MICs of the extracts and essential oil were determined by the broth microdilution method in sterile 96‐well microplates using cation‐adjusted Mueller–Hinton broth (CAMHB) [35]. Prior to testing, all preparations were dissolved in 5% (*v*/*v*) DMSO and then serially diluted in CAMHB. For the essential oil, the material was weighed directly before preparing the test solutions; therefore, the values reported in this study represent final mass concentrations in the assay wells (mg/mL). The concentration range used in this study was 0.011, 0.11, 1.11, and 11.11 mg/mL. This broad screening range was selected to compare the antibacterial activity of the tested plant preparations across the clinical isolate set, rather than to use a conventional, closely spaced twofold dilution series. Each well was inoculated with bacterial suspensions adjusted to approximately 5 × 10^5^ CFU/mL and incubated at 37°C for 24 h.

The MIC was defined as the lowest concentration that completely inhibited visible bacterial growth. The MBC was determined by subculturing 10 *μ*L aliquots from wells with no visible growth onto MHA plates and incubating the plates at 37°C for 24 h. The lowest concentration from which no colony growth was recovered was considered the MBC under the operational conditions of this study. No antibiotic‐positive reference control was included in this assay, and no formal quality‐control testing was performed.

### 2.8. Phytochemical Analysis Using HPLC

Phenolic constituents in the plant extracts were qualitatively profiled by high‐performance liquid chromatography (HPLC). The analysis was performed on a reversed‐phase C18 column (250 × 4.6 mm, 5 *μ*m particle size) at ambient temperature. The mobile phase consisted of water containing 0.1% formic acid (solvent A) and acetonitrile (solvent B), delivered at a constant flow rate of 1 mL/min. The injection volume for both samples and reference standards was 20 *μ*L. Chromatograms were recorded with a UV–Vis detector at 280 nm for catechin and at 320 nm for chlorogenic acid and quercetin. Reference standards of catechin, chlorogenic acid, and quercetin were prepared in HPLC‐grade methanol and analyzed under identical chromatographic conditions. Retention times and peak areas were recorded using the instrument software. Peaks in the extract chromatograms were compared with the corresponding reference standards by retention‐time matching for qualitative screening. Although peak‐area data were recorded, the concentrations of catechin, chlorogenic acid, and quercetin were not calculated because multipoint calibration curves had not been established. Accordingly, the HPLC analysis was conducted as a qualitative screening procedure [36].

### 2.9. Gas Chromatography–Mass Spectrometry (GC–MS) Analysis of Essential Oils

The chromatographic profile of the essential oil was evaluated by GC–MS. Analyses were performed on a GC–MS system equipped with an HP‐5MS capillary column (30 m × 0.25 mm internal diameter, 0.25 *μ*m film thickness). Helium served as the carrier gas at a constant flow rate of 1 mL/min. The oven temperature was programmed from 60°C to 280°C at 4°C/min. The injector temperature was maintained at 250°C, and electron ionization was performed at 70 eV. Acquired mass spectra were compared with entries in the NIST 14 mass spectral library, and the resulting library‐search outputs were retained for descriptive reference only [37]. Individual peaks were not interpreted as confirmed compound identifications.

### 2.10. Statistical Analysis

Because the same 91 *E. coli* isolates were evaluated against all five‐plant preparations, MIC and MBC values were analyzed as paired ordinal outcomes. Observations exceeding the highest tested concentration (> 11.11 mg/mL) were coded as 110.11 mg/mL for rank‐based analysis. Overall differences among the five preparations were assessed using Friedman′s test, followed by pairwise Wilcoxon signed‐rank tests with Holm adjustment for multiple comparisons. Exact *p* values were reported, and two‐sided adjusted *p* < 0.05 was considered statistically significant.

## 3. Results

The antimicrobial susceptibility profiles of 91 phenotypically MDR urinary *E. coli* isolates to 18 antimicrobial agents are summarized in Figure 1. All isolates met the operational MDR criterion, defined as nonsusceptibility to at least one antimicrobial agent in three or more antimicrobial classes. The highest susceptibility rates were observed for MEM (84.9%), IMP (74.2%), and PIP (73.1%). In contrast, susceptibility to AMP, AMX, and OXA was very low. These findings indicate resistance or nonsusceptibility across multiple antimicrobial classes, whereas carbapenems and PIP retained comparatively higher in vitro activity against the tested isolates.

**Figure 1:**
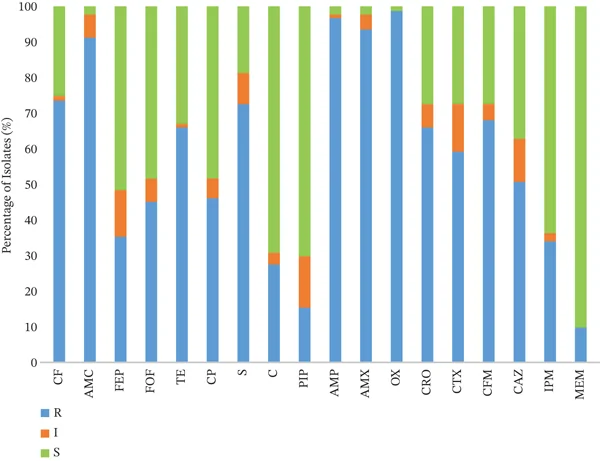
Antimicrobial susceptibility profiles of 91 MDR *E. coli* isolates across 18 antimicrobial agents. Data are presented as the cumulative percentages of resistant (R), intermediate (I), and susceptible (S) isolates according to CLSI breakpoints.

The antibacterial activity of the five plant‐derived preparations—EoFs, EtFs, AqFs, EtEc, and AqEc—was initially screened using the agar well diffusion method. This preliminary screening revealed variable activity and heterogeneous responses among the 91 phenotypically MDR urinary *E. coli* isolates. Figure 2 presents a heatmap of inhibition zone results across the tested concentrations. For visualization purposes only, isolates with no detectable inhibition zone against a particular preparation were omitted from the corresponding heatmap column. The numbers of isolates without a detectable inhibition zone were 1, 10, 17, 19, and 32 for EoFs, EtFs, AqFs, EtEc, and AqEc, respectively. These graphical omissions were made solely to improve readability and did not alter the underlying isolate‐level dataset.

**Figure 2:**
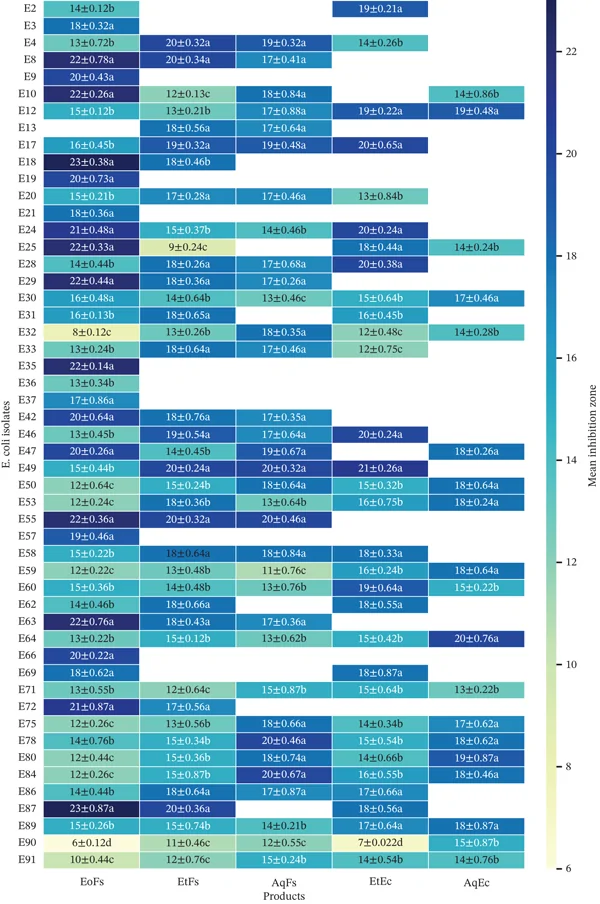
Heatmap of inhibition zone diameters produced by the five plant‐derived preparations—EoFs, EtFs, AqFs, EtEc, and AqEc—against phenotypically MDR urinary *E. coli* isolates at the tested concentration levels. The letters a–d indicate the tested concentration levels, from the highest to the lowest concentration. Blank cells represent isolates with no detectable inhibition zone for the respective preparation and were omitted from the visualization solely to improve readability. These omissions do not represent missing values in the underlying dataset.

As shown in Figure 3, EoFs exhibited the broadest inhibitory profile, with cumulative inhibition of 54% (49 of 91 isolates) across the tested concentration range (0.011–11.11 mg/mL). At the highest concentration of 11.11 mg/mL, 27% (25 of 91 isolates) were inhibited, and 16% (15 of 91 isolates) were killed by EoFs. The overall antibacterial activity followed the descriptive trend: EoFs > EtFs > EtEc > AqFs > AqEc, suggesting that the essential oil and ethanolic preparations generally exhibited greater in vitro inhibitory activity than the aqueous extracts. This pattern is descriptive and should not be interpreted as evidence that all pairwise differences among preparations were statistically significant.

**Figure 3:**
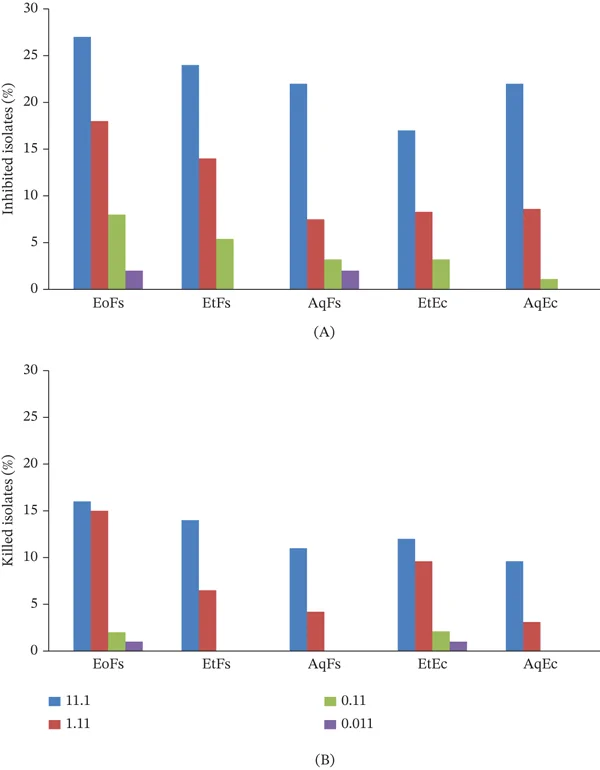
Distribution of inhibition and killing endpoints among the tested *E. coli* isolates. (A) Percentage of isolates with inhibition endpoints and (B) percentage of isolates with killing endpoints at 11.11, 1.11, 0.11, and 0.011 mg/mL for each plant preparation. Bars represent concentration‐specific endpoint categories and should not be interpreted as cumulative activity at those concentrations. Where reported, “total” denotes the cumulative percentage of isolates showing an endpoint within the tested concentration range.

The antimicrobial activity of the five preparations against the 91 phenotypically MDR urinary *E. coli* isolates was further assessed by determining MICs and MBCs. The frequency distributions of MIC and MBC values across the tested concentration categories are shown in Figure 4. A considerable proportion of MIC and MBC observations exceeded the highest tested concentration (> 11.11 mg/mL). For rank‐based analyses, these right‐censored observations were coded as 110.11 mg/mL and treated as upper limits rather than exact MIC or MBC concentrations.

**Figure 4:**
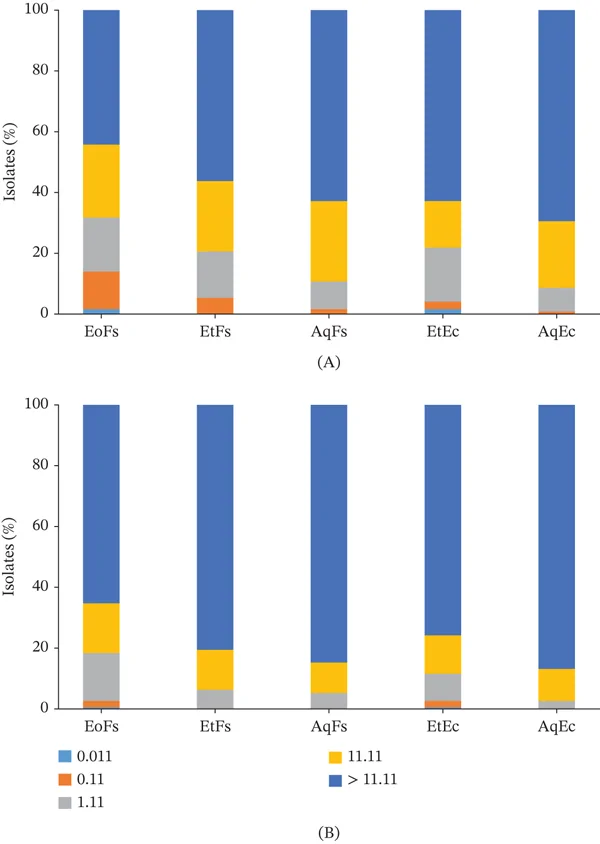
Distribution of (A) MIC and (B) MBC values of the five tested plant preparations against 91 phenotypically MDR urinary *E. coli* isolates. Data are presented as percentages (*n* = 91 paired observations per preparation). Values > 11.11 mg/mL were coded as 110.11 mg/mL for rank‐based analysis. MIC and MBC were compared separately using Friedman′s test followed by two‐sided Wilcoxon signed‐rank tests with Holm adjustment.

Friedman′s test showed significant overall differences among the five preparations for both MIC (*χ* ^2^(4) = 35.734, *p* = 3.28 × 10^−7^, Kendall′s *W* = 0.098) and MBC (*χ* ^2^(4) = 37.575, *p* = 1.37 × 10^−7^, Kendall′s *W* = 0.103) values. Holm‐adjusted pairwise Wilcoxon signed‐rank tests indicated that EoFs had lower MIC values than EtFs (*p* = 0.0001), AqFs (*p* < 0.001), and AqEc (*p* = 0.0007). EtFs also had lower MIC values than AqFs (*p* = 0.0029), whereas EtEc had lower MIC values than AqEc (*p* < 0.001). For MBC, EoFs had lower values than EtFs (*p* = 0.0001), AqFs (*p* < 0.001), and AqEc (*p* = 0.0004), whereas EtEc had lower MBC values than AqEc (*p* = 0.0008). Despite the statistical significance of the overall tests, the small Kendall′s W values indicate a small overall effect magnitude.

RP‐HPLC analysis of the extracts was conducted as a qualitative screen for selected phenolic compounds by comparing sample retention times with those of the corresponding authentic reference standards (Figures 5, 6, and 7). Chromatograms recorded at 280 nm showed peaks with retention times comparable to the catechin standard in some preparations, whereas chromatograms recorded at 320 nm showed peaks with retention times comparable with the quercetin standard in some preparations. No peak with a retention time comparable with the chlorogenic acid standard was observed under the chromatographic conditions used. Because the assessment was qualitative and the chromatograms had different vertical scales, quantitative comparisons of compound levels among extraction solvents or plant species were not performed.

**Figure 5:**
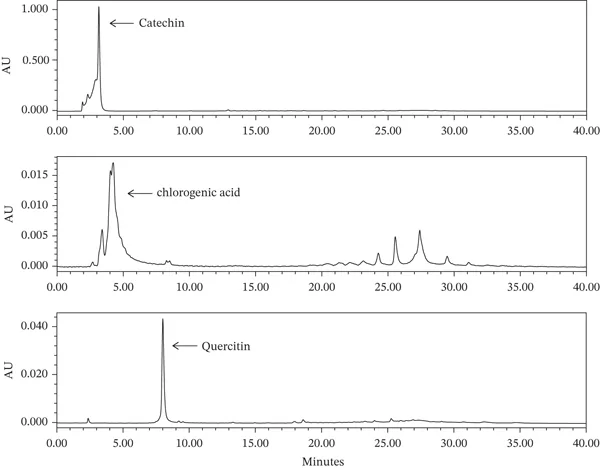
RP‐HPLC chromatograms of the reference standards. Catechin was detected at 280 nm, whereas chlorogenic acid and quercetin were detected at 320 nm. Reference standards were injected to establish retention times for descriptive retention‐time matching with extract profiles.

**Figure 6:**
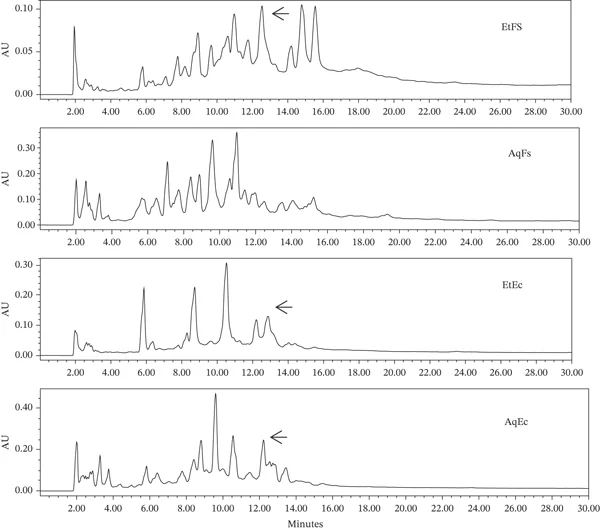
RP‐HPLC chromatograms of the plant extracts—EtFs, AqFs, EtEc, and AqEc—recorded at 280 nm. The chromatograms were used to qualitatively compare extract profiles with the catechin reference standard based on retention time.

**Figure 7:**
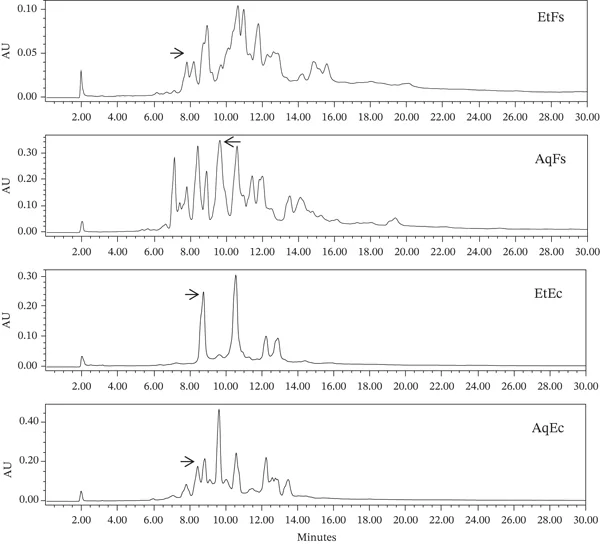
RP‐HPLC chromatograms of the plant extracts—EtFs, AqFs, EtEc, and AqEc—recorded at 320 nm. The chromatograms were used to qualitatively compare extract profiles with the chlorogenic acid and quercetin reference.

The chromatographic fingerprint of EoFs, which exhibited the broadest antibacterial profile among the tested preparations, was evaluated by GC–MS (Figure 8 and Table 1). The analysis produced a total ion chromatogram with multiple resolved and unresolved peaks. Electron‐ionization mass spectra of selected peaks were compared with entries in the NIST 14 spectral library for descriptive purposes only. Because experimentally determined retention indices and authentic reference standards were unavailable, the library‐search outputs were not interpreted as confirmed or tentative compound identifications. Accordingly, the GC–MS findings are presented as an unresolved qualitative chromatographic fingerprint rather than as a quantitative phytochemical characterization. No specific chromatographic feature was linked to the observed antibacterial activity, and the chemical basis and mechanisms underlying this activity remain to be determined in future studies.

**Figure 8:**
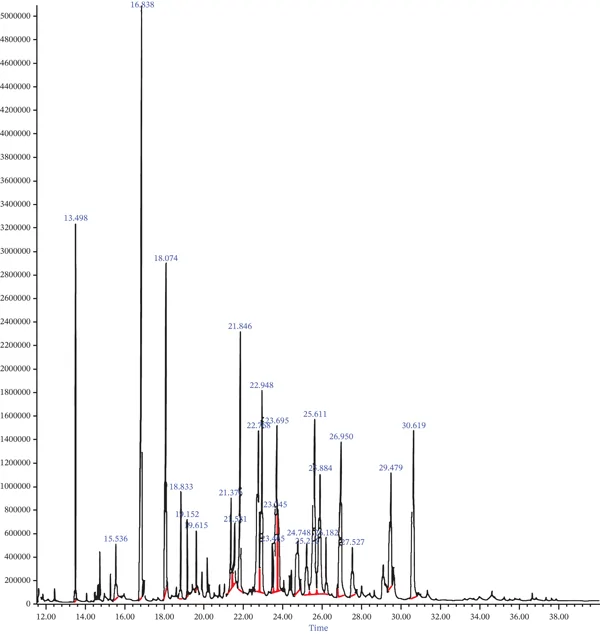
GC–MS total ion chromatogram of the EoFs. Numbers indicate the retention times (min) of selected prominent peaks. Library comparisons with the NIST 14 database are shown for descriptive purposes only and do not constitute confirmed compound identifications; therefore, the chromatogram is interpreted as a qualitative chromatographic fingerprint.

**Table 1:** Descriptive GC–MS chromatographic peak features and unverified NIST library‐search outputs for the EoFs.

Peak no.	Compounds	RT	Area (%)	Probability (%)
1	Benzenemethanol, 4‐(1‐methylethyl)	13.48	5.78	91
2	Benzenemethanol, *α*,*α*‐dimethyl‐	15.53	1.43	91
3	(−)‐(1S,5R)‐endo‐2 (R),8,8‐trimethylbicyclo[3.2.1]octan‐3‐one	16.83	14.33	64
4	3‐furaldehyde	18.07	7.46	30
5	Linalool	18.83	2.03	42
6	Phenol, 5‐methyl‐2‐(1‐methylethyl)	19.15	1.19	95
7	2,5‐dimethyl‐4‐vinylhex‐5‐en‐2,3‐diol	19.61	0.88	28
8	6‐methylene‐3‐oxabicyclo[3.3.0]octan‐2‐one	21.37	2.21	72
9	O‐menthan‐8‐ol	21.56	1.64	47
10	1‐methylbicyclo[2.2.1]hepta‐2‐ene	21.84	6.12	49
11	(1RS,2RS,4RS)‐2‐propylbicyclo[2.2.2]oct‐5‐en‐2‐ol	22.76	6.44	64
12	1‐methoxy‐1,3‐cyclohexadiene	22.94	5.56	78
13	2,3,5‐trimethylfuran	23.46	0.81	35
14	1‐methoxy‐1,3‐cyclohexadiene	23.69	5.79	72
15	Cyclopentanone, 3‐methyl‐2‐(2‐pentenyl)‐	23.74	2.65	27
16	Furan, 2,3‐dihydro‐4‐methyl	24.74	2.52	27
17	1,4‐pentadiene, 2,3,3‐trimethyl	25.21	1.99	38
18	2,3,3‐trimethyl‐1‐hexene	25.61	7.03	27
19	1‐pentanone, 1‐(3‐furanyl)‐4‐hydroxy‐	25.88	5.51	43
20	Hydroxy citronellal	26.18	1.28	38
21	Cyclohexanol, 1‐methyl‐4‐(1‐methylethyl)‐	26.95	5.83	47
22	(1R,2S)‐2‐acetyl‐1‐methylcyclobutaneacetic acid	27.52	1.77	22
23	2‐cyclopentene‐1‐thione, 3‐methyl‐	29.47	3.92	58
24	Bicyclo[3.1.1]heptane, 2,6,6‐trimethyl‐, [1R‐(1*α*,2*α*,5*α*)]‐	30.61	5.70	38
Total			99.87	

*Note:* The listed names are unverified NIST 14 library‐search outputs provided for descriptive reference only and do not constitute compound identifications. Relative peak areas are reported as descriptive chromatographic features.

## 4. Discussion

UTIs are among the most common bacterial infections worldwide, and *E. coli* remains the leading pathogen in both community‐ and hospital‐acquired cases. The rising prevalence of MDR *E. coli* is clinically concerning because resistance to commonly prescribed antibiotics can lead to treatment failure, recurrent infection, prolonged hospitalization, and increased healthcare costs [2, 3]. In this context, plant‐derived antimicrobial agents warrant investigation as potential sources of novel antibacterial compounds. Apiaceae species are particularly relevant because they contain diverse secondary metabolites, including terpenoids, flavonoids, coumarins, and phenolic compounds, several of which have been linked to antimicrobial activity in prior studies [15, 26–28]. Accordingly, evaluating *F. subpinnata* and *E. caucasicum* against MDR urinary *E. coli* isolates provides clinically relevant preliminary evidence supporting their potential as sources of antibacterial agents.

All 91 urinary *E. coli* isolates in this study were resistant to at least three antibiotic classes and were therefore classified as MDR. This uniformly resistant phenotype provides a stringent in vitro setting for assessing the activity of the plant‐derived preparations tested. Among the tested antibiotics, MEM showed the highest activity, followed by IMP and PIP. This pattern is consistent with reports that carbapenems often retain activity against MDR Gram‐negative bacilli. However, the reduced activity of IMP compared with MEM and the limited activity of most other *β*‐lactams underscore the limited therapeutic options available for these isolates. Carbapenem resistance is increasingly reported in many regions, further underscoring the importance of identifying complementary antibacterial strategies [4, 38]. The observed *β*‐lactam resistance pattern may reflect mechanisms reported in urinary *E. coli*, including extended‐spectrum *β*‐lactamases, AmpC enzymes, altered porin expression, and efflux pump overactivity [39]. However, these mechanisms were not investigated in this study and should not be inferred directly from the phenotypic susceptibility results.

Against this MDR background, the plant‐derived preparations demonstrated measurable inhibitory and bactericidal activity, although the magnitude varied substantially across preparations and isolates. EoFs showed the most consistent overall activity, producing the largest inhibition zones in the screening assay and exhibiting the most favorable MIC and MBC distributions across the tested concentration range. The overall cumulative inhibitory activity pattern was EoFs > EtFs > EtEc > AqFs > AqEc, and cumulative bactericidal activity followed a broadly similar trend. Importantly, the apparent superiority of EoFs was not based solely on agar diffusion results; it was also supported by MIC and MBC distributions obtained by broth microdilution. Nonetheless, although Friedman′s analysis confirmed statistically significant differences among the preparations, the calculated Kendall′s *W* effect sizes were small (*W* ≈ 0.10). Therefore, this ranking should be interpreted as a general descriptive trend rather than an absolute, uniformly strong superiority across all isolates tested under the in vitro conditions.

Nevertheless, the activity of all preparations was heterogeneous across the 91 isolates. MIC and MBC values spanned the tested concentration categories, from 0.011 mg/mL to > 11.11 mg/mL, with a substantial proportion of isolates falling into the highest category. This pattern indicates reduced susceptibility in a substantial subset of isolates across the tested concentration range, rather than a complete lack of activity. Because observations in the > 11.11 mg/mL category were right‐censored, the exact MIC or MBC values for these isolates could not be determined. Therefore, the results should be interpreted primarily as distributions of inhibitory and bactericidal activity within the tested concentration range, rather than as precise estimates of central MIC or MBC values. The variability among isolates may reflect differences in bacterial envelope permeability, biofilm‐associated tolerance, efflux‐related activity, and other intrinsic or acquired resistance‐associated characteristics reported in MDR *E. coli* [40]; however, these factors were not directly assessed in the present study.

The stronger activity of the essential oil, particularly EoFs, relative to the ethanolic and aqueous fractions may reflect differences in the physicochemical composition of these preparations. Essential oils are generally enriched in volatile and lipophilic constituents that may interact with bacterial membranes and alter their permeability [41]. Such properties could facilitate antibacterial activity against Gram‐negative bacteria, whose outer membrane commonly limits the entry of many antimicrobial compounds. However, membrane disruption or any other specific mechanism was not experimentally examined in the present study. Therefore, these literature‐based explanations should not be interpreted as direct mechanistic findings of our work. In contrast, aqueous extracts are expected to contain relatively more polar constituents, which may differ in membrane affinity, stability, and ability to reach intracellular bacterial targets. The greater activity of ethanolic fractions compared with aqueous fractions further suggests that the extraction solvent influences the recovery and relative concentrations of bioactive phytochemicals. Similar solvent‐dependent differences in antibacterial activity have been reported in plant‐based antimicrobial studies [42].

The present results should be interpreted in relation to, rather than merely alongside, previous studies of Apiaceae plants. Essential oils from *E. caucasicum* and *Caucalis platycarpos* have previously shown inhibitory activity against gram‐negative bacteria, including *E. coli* [43]. Likewise, antimicrobial activity has been reported for essential oils or metabolites of *Eryngium billardieri* and *Eryngium pristis* [15, 44]. These studies support the broader antibacterial potential of Apiaceae taxa, but direct comparisons of potency should be made cautiously because plant chemotypes, geographic origin, plant part, harvesting conditions, extraction methods, bacterial strains, and susceptibility‐testing procedures may differ substantially among studies. Previous reports that methanolic extracts of *F. subpinnata* inhibited *E. coli* at MIC values of approximately 1 mg/mL [40, 45] are broadly consistent with the susceptibility observed for some isolate–preparation combinations in the current study. However, the wide distribution of MIC and MBC categories observed here also indicates that activity against clinical MDR urinary isolates cannot be adequately represented by a single value obtained using different bacterial strains or plant preparations. Reports on *Eryngium planum*, *Eryngium campestre*, and *Eryngium maritimum* further support the genus′s antimicrobial potential while illustrating the substantial phytochemical and biological variability among *Eryngium* species [44].

Differences between *F. subpinnata* and *E. caucasicum* preparations likely stem from both species‐specific phytochemical variation and extraction‐related factors. Even among closely related taxa, the qualitative and quantitative profiles of terpenes, flavonoids, phenolic acids, coumarins, and polyacetylenes can vary considerably. In crude preparations, antibacterial effects may arise from the combined activity of multiple constituents rather than a single dominant compound. Thus, the greater activity of EoFs may reflect a more favorable composition, concentration, or interaction among bioactive constituents. This possibility aligns with the observed pattern but cannot be confirmed without bioassay‐guided fractionation, characterization of active constituents, and direct testing of isolated compounds.

An important aspect of the present findings is that antibacterial activity was observed against isolates with phenotypically confirmed MDR profiles. However, plant preparations and conventional antibiotics should not be considered directly equivalent in potency or clinical utility. The current data provide evidence of in vitro activity in an MDR background, rather than evidence that these products can overcome defined antibiotic‐resistance mechanisms or serve as a therapeutic alternative to established antibiotics. Although previous studies suggest that essential oil constituents may disrupt membranes or alter permeability, these mechanisms were not experimentally investigated in the present work. Such effects remain hypotheses based on the literature regarding the observed activity of *F. subpinnata* [40, 45]. Similarly, the potential for additive or synergistic effects between the tested preparations and conventional antibiotics, as well as the possibility of resistance development after repeated exposure, remains unknown and requires dedicated investigation.

Several limitations should be considered when interpreting these findings. First, antibacterial activity was evaluated exclusively in vitro and therefore cannot be directly extrapolated to clinical efficacy in patients with UTIs.

Second, the isolates were obtained retrospectively from routine diagnostic urine specimens collected from patients with suspected UTI. However, detailed clinical information and quantitative bacteriuria thresholds (CFU/mL) were unavailable for the archived samples. Consequently, symptomatic UTI could not be distinguished from asymptomatic bacteriuria at the patient level. In addition, the community‐ or hospital‐acquired origin of the infections could not be determined, and it was not possible to establish whether multiple isolates originated from the same patient. Accordingly, the organisms are more appropriately described as phenotypically MDR urinary *E. coli* isolates rather than fully characterized uropathogenic strains.

Third, the isolates were identified using standard microbiological and biochemical procedures and classified as MDR based on antimicrobial susceptibility testing. Molecular and pathotypic characterization was not performed. Therefore, ESBL production, carbapenemase activity, phylogenetic group, specific resistance determinants, biofilm formation, and virulence‐associated genes were not assessed.

Fourth, agar well diffusion was used as an initial screening method. This technique has inherent limitations for hydrophobic and volatile constituents, including essential‐oil components, because these compounds may not diffuse uniformly through agar. Therefore, inhibition‐zone diameters should be interpreted with caution, with greater emphasis on broth microdilution findings.

Fifth, the MIC and MBC assays used a broad concentration range rather than a conventional, closely spaced twofold dilution series. Therefore, the exact inhibitory and bactericidal thresholds could be estimated only within the tested range. In addition, the broth microdilution experiments did not include an antibiotic‐positive reference control or formal quality‐control testing. The MBC endpoint was operationally defined as the absence of visible colony growth after subculturing 10 *μ*L from wells that showed no visible growth.

Sixth, observations exceeding 11.11 mg/mL were right‐censored at the upper assay limit and coded as 110.11 mg/mL for rank‐based analysis. This coding preserved the ordinal ordering of the observations but did not allow exact MIC or MBC values above the assay limit to be distinguished. Accordingly, the Friedman and pairwise analyses should be interpreted as analyses of coded ordinal outcomes rather than of uncensored continuous concentrations.

Finally, the GC–MS analysis was limited to chromatographic fingerprinting and unverified library‐search results. Retention indices were not determined experimentally, and authentic reference standards were not used for confirmation. Therefore, individual chromatographic peaks should not be interpreted as confirmed compound identifications, and the contribution of specific chemical constituents to the observed antibacterial activity could not be established. The active constituents were neither isolated nor tested individually. In addition, toxicity, pharmacokinetic behavior, mechanisms of action, and in vivo efficacy were not assessed.

The plant preparations were derived from a single plant collection and a single extraction or hydrodistillation batch. The replicate measurements were technical replicates only and did not represent independent biological replicates. Consequently, biological reproducibility across independent plant collections or preparation batches was not demonstrated, and variation associated with collection site, season, plant chemotype, and extraction or hydrodistillation batch was not evaluated. Moreover, because the isolates originated from a specific geographic and clinical setting, the generalizability of the findings to other populations and regions may be limited.

Future investigations should employ more rigorous phytochemical standardization using GC‐MS, HPLC, and/or LC‐MS/MS, supported by experimentally determined retention indices and authentic reference standards. Bioassay‐guided fractionation would be especially valuable for determining whether antibacterial activity is attributable to individual dominant constituents or to synergistic interactions among multiple compounds. Evaluating combinations with conventional antibiotics, particularly *β*‐lactams and carbapenems, may also clarify whether these preparations have potential as adjunctive rather than stand‐alone antibacterial agents against MDR *E. coli*. In addition, toxicity assessment, pharmacokinetic studies, formulation development, and appropriately designed in vivo infection models are necessary before any therapeutic application can be considered. Overall, *F. subpinnata*, particularly its essential oil, showed promising in vitro antibacterial activity against phenotypically MDR urinary *E. coli* isolates; however, its therapeutic relevance remains to be established through comprehensive phytochemical, mechanistic, toxicological, and in vivo studies.

## 5. Conclusion

The high prevalence of phenotypically MDR urinary *E. coli* isolates highlights the continuing challenge of treating infections caused by antimicrobial‐resistant pathogens. Among the tested plant‐derived preparations, the essential oil of *F. subpinnata* exhibited the broadest in vitro antibacterial profile, including both inhibitory and killing activity, whereas the ethanolic fractions showed intermediate activity and the aqueous preparations were generally less active. These findings support further investigation of *F. subpinnata* essential oil as a source of plant‐derived antibacterial activity; however, the results remain limited to the in vitro conditions of this study and do not establish clinical efficacy or therapeutic equivalence to conventional antibiotics.

**Nomenclature**
AqEc: aqueous extract of *E. caucasicum*
AqFs: aqueous extract of *F. subpinnata*
*E. caucasicum*
: *Eryngium caucasicum*
*E. coli*
: *Escherichia coli*
EoFs: essential oil of *F. subpinnata*
EtEc: ethanolic extract of *E. caucasicum*
EtFs: ethanolic extract of *F. subpinnata*
*F. subpinnata*
: *Froriepia subpinnata*
GC–MS: gas chromatography–mass spectrometry
HPLC: high‐performance liquid chromatography
MBC: minimum bactericidal concentration
MDR: multidrug‐resistant
MHA: Mueller–Hinton agar
MIC: minimum inhibitory concentration
UPEC: uropathogenic *Escherichia coli*
UTI: urinary tract infection

## Author Contributions

**M.A.:** conceptualization, data collection, sample analysis, and writing—original draft. **M.J.M.M.** and **D.B.:** supervision, methodology, and writing—review and editing. **M.A.**, **M.J.M.M.**, and **D.B.:** data analysis, validation, writing, review, and editing. **M.J.M.M.** and **D.B.:** project administration, supervision, and critically revised the manuscript.

## Funding

No funding was received for this manuscript.

## Conflicts of Interest

The authors declare no conflicts of interest.

## Supporting Information

Additional supporting information can be found online in the Supporting Information section.

## Supporting information


**Supporting Information 1** File S1: Agar well diffusion data (inhibition zone measurements across isolates).

**Supporting Information 2** File S2: HPLC chromatographic data and integration sheets for plant extracts and standards.

**Supporting Information 3** File S3: MIC/MBC datasets and complete antibiotic susceptibility profiles for all 91 MDR *E. coli* clinical isolates.

## Data Availability

The data that support the findings of this study are available from the corresponding author upon reasonable request.
