# Supplementary material for: Comparative Evaluation of the In Vitro Antibacterial Activity of Preliminary Plant Preparations Against Phenotypically Multidrug‐Resistant Urinary Isolates of Escherichia Coli
Source: Biomed Res Int. 2026 Sep 30;2026:1850504. doi: 10.1155/bmri/1850504 (PMC13627857; doi:10.1155/bmri/1850504)
Supplement: Supplementary file 1 [file BMRI-2026-1850504-s001.docx]

**Table 2.** Antimicrobial activity of the plant extracts evaluated by the agar well diffusion method.

|  | **Mean diameter of growth inhibition zone± standard deviation (mm)** | | | | |
| --- | --- | --- | --- | --- | --- |
| ***E. coli* isolates** | EoFs | EtFs | AqFs | EtEc | AqEc |
| E1 | - | - | - | - | - |
| E2 | 14±0.12^b^ | - | - | 19±0.21^a^ | - |
| E3 | 18±0.32^a^ | - | - | - | - |
| E4 | 13±0.72^b^ | 20±0.32^a^ | 19±0.32^a^ | 14±0.26^b^ | - |
| E5 | - | - | - | - | - |
| E6 | - | - | - | - | - |
| E7 | - | - | - | - | - |
| E8 | 22±0.78^a^ | 20±0.34^a^ | 17±041^a^ | - | - |
| E9 | 20±043^a^ | - | - | - | - |
| E10 | 22±0.26^a^ | 12±0.13^c^ | 18±0.84^a^ | - | 14±0.86^b^ |
| E11 | - | - | - | - | - |
| E12 | 15±0.12 ^b^ | 13±0.21^b^ | 17±0.88 ^a^ | 19±0.22 ^a^ | 19±0.48^a^ |
| E13 | - | 18±0.56 ^a^ | 17±0.64^a^ | - | - |
| E14 | - | - | - | - | - |
| E15 | - | - | - | - | - |
| E16 | - | - | - | - | - |
| E17 | 16±0.45 ^b^ | 19±0.32^a^ | 19±0.48^a^ | 20±0.65^a^ | - |
| E18 | 23±0.38 ^a^ | 18±0.46^b^ | - | - | - |
| E19 | 20±0.73 ^a^ | - | - | - | - |
| E20 | 15±0.21 ^b^ | 17±0.28^a^ | 17±0.46^a^ | 13±0.84^b^ | - |
| E21 | 18±0.36 ^a^ | - | - | - | - |
| E22 | - | - | - | - | - |
| E23 | - | - | - | - | - |
| E24 | 21±0.48^a^ | 15±0.37^b^ | 14±0.46^b^ | 20±0.24^a^ | - |
| E25 | 22±0.33^a^ | 9±0.24^c^ | - | 18±0.44^a^ | 14±0.24^b^ |
| E26 | - | - | - | - | - |
| E27 | - | - | - | - | - |
| E28 | 14±0.44^b^ | 18±0.26^a^ | 17±0.68^a^ | 20±0.38^a^ | - |
| E29 | 22±0.44^a^ | 18±0.36^a^ | 17±0.26^a^ | - | - |
| E30 | 16±0.48^a^ | 14±0.64^b^ | 13±0.46^c^ | 15±0.64^b^ | 17±0.46^a^ |
| E31 | 16±0.13^b^ | 18±0.65^a^ | - | 16±0.45^b^ | - |
| E32 | 8±0.12^c^ | 13±0.26^b^ | 18±0.35^a^ | 12±0.48^c^ | 14±0.28^b^ |
| E33 | 13±0.24^b^ | 18±0.64^a^ | 17±0.46^a^ | 12±0.75^c^ | - |
| E34 | - | - | - | - | - |
| E35 | 22±0.14^a^ | - | - | - | - |
| E36 | 13±0.34^b^ | - | - | - | - |
| E37 | 17±0.86 ^a^ | - | - | - | - |
| E38 | - | - | - | - | - |
| E39 | - | - | - | - | - |
| E40 | - | - | - | - | - |
| E41 | - | - | - | - | - |
| E42 | 20±0.64^a^ | 18±0.76^a^ | 17±0.35^a^ | - | - |
| E43 | - | - | - | - | - |
| E44 | - | - | - | - | - |
| E45 | - | - | - | - | - |
| E46 | 13±0.45^b^ | 19±0.54^a^ | 17±0.64^a^ | 20±0.24^a^ | - |
| E47 | 20±0.26^a^ | 14±0.45^b^ | 19±0.67^a^ | - | 18±0.26^a^ |
| E48 | - | - | - | - | - |
| E49 | 15±0.44 ^b^ | 20±0.24^a^ | 20±0.32^a^ | 21±0.26 ^a^ | - |
| E50 | 12±0.64^c^ | 15±0.24 ^b^ | 18±0.64^a^ | 15±0.32^b^ | 18±0.64^a^ |
| E51 | - | - | - | - | - |
| E52 | - | - | - | - | - |
| E53 | 12±0.24^c^ | 18±0.36^b^ | 13±0.64^b^ | 16±0.75^b^ | 18±0.24^a^ |
| E54 | - | - | - | - | - |
| E55 | 22±0.36^a^ | 20±0.32^a^ | 20±0.46^a^ | - | - |
| E56 | - | - | - | - | - |
| E57 | 19±0.46^a^ | - | - | - | - |
| E58 | 15±0.22^b^ | 18±0.64^a^ | 18±0.84^a^ | 18±0.33^a^ | - |
| E59 | 12±0.22^c^ | 13±0.48^b^ | 11±0.76^c^ | 16±0.24^b^ | 18±0.64^a^ |
| E60 | 15±0.36^b^ | 14±0.48^b^ | 13±0.76^b^ | 19±0.64^a^ | 15±0.22^b^ |
| E61 | - | - | - | - | - |
| E62 | 14±0.46 ^b^ | 18±0.66 ^a^ | - | 18±0.55^a^ | - |
| E63 | 22±0.76 ^a^ | 18±0.43 ^a^ | 17±0.36 ^a^ | - | - |
| E64 | 13±0.22 ^b^ | 15±0.12 ^b^ | 13±0.62 ^b^ | 15±0.42^b^ | 20±0.76^a^ |
| E65 | - | - | - | - | - |
| E66 | 20±0.22^a^ | - | - | - | - |
| E67 | - | - | - | - | - |
| E68 | - | - | - | - | - |
| E69 | 18±0.62^a^ | - | - | 18 ±0.87^a^ | - |
| E70 | - | - | - | - | - |
| E71 | 13±0.55^b^ | 12±0.64^c^ | 15±0.87^b^ | 15±0.64^b^ | 13±0.22^b^ |
| E72 | 21±0.87^a^ | 17±0.56^a^ | - | - | - |
| E73 | - | - | - | - | - |
| E74 | - | - | - | - | - |
| E75 | 12±0.26^c^ | 13±0.56^b^ | 18±0.66^a^ | 14±0.34^b^ | 17±0.62^a^ |
| E76 | - | - | - | - | - |
| E77 | - | - | - | - | - |
| E78 | 14±0.76^b^ | 15±0.34^b^ | 20±0.46^a^ | 15±0.54^b^ | 18±0.62^a^ |
| E79 | - | - | - | - | - |
| E80 | 12±0.44^c^ | 15±0.36^b^ | 18±0.74^a^ | 14±0.66^b^ | 19±0.87^a^ |
| E81 | - | - | - | - | - |
| E82 | - | - | - | - | - |
| E83 | - | - | - | - | - |
| E84 | 12±0.26^c^ | 15±0.87^b^ | 20±0.67^a^ | 16±0.55^b^ | 18±0.46^a^ |
| E85 | - | - | - | - | - |
| E86 | 14±0.44^b^ | 18±0.64^a^ | 17±0.87^a^ | 17±0.66^a^ | - |
| E87 | 23±0.87^a^ | 20±0.36^a^ | - | 18±0.56^a^ | - |
| E88 | - | - | - | - | - |
| E89 | 15±0.26^b^ | 15±0.74^b^ | 14±0.21^b^ | 17±0.64^a^ | 18±0.87^a^ |
| E90 | 6±0.12^d^ | 11±0.46^c^ | 12±0.55^c^ | 7±0.022^d^ | 15±0.87^b^ |
| E91 | 10±0.44^c^ | 12±0.76^c^ | 15±0.24^b^ | 14±0.54^b^ | 14±0.76^b^ |

EoFs: essential oil of *Eryngium caucasicum*; EtFs: ethanolic extract of *Froriepia subpinnata*; AqFs: aqueous extract of *Froriepia subpinnata*; EtEc: ethanolic extract of *Eryngium caucasicum*; AqEc: aqueous extract of *Eryngium caucasicum*). The letters a, b, c and d indicate concentrations of 11.11, 1.11, 0.11 and 0.011 mg/ml, respectively.
